# The Molecular and Genetic Characterization of Second Chromosome Balancers in *Drosophila melanogaster*

**DOI:** 10.1534/g3.118.200021

**Published:** 2018-02-02

**Authors:** Danny E. Miller, Kevin R. Cook, Elizabeth A. Hemenway, Vivienne Fang, Angela L. Miller, Karen G. Hales, R. Scott Hawley

**Affiliations:** * Stowers Institute for Medical Research, Kansas City, Missouri 64110,; † MD-PhD Physician–Scientist Training Program and; ** Department of Molecular and Integrative Physiology, University of Kansas Medical Center, Kansas City, Kansas 66160; ‡ Department of Biology, Indiana University, Bloomington, Indiana 47405, and; § Department of Biology, Davidson College, North Carolina 28035

**Keywords:** balancer chromosomes, inversion breakpoints, meiosis, whole-genome sequencing, crossing over

## Abstract

Balancer chromosomes are multiply inverted and rearranged chromosomes used in *Drosophila melanogaster* for many tasks, such as maintaining mutant alleles in stock and complex stock construction. Balancers were created before molecular characterization of their breakpoints was possible, so the precise locations of many of these breakpoints are unknown. Here, we report or confirm the positions of the 14 euchromatic breakpoints on the *2^nd^* chromosome balancers *SM1*, *SM5*, *CyO*, and *SM6a*. This total includes three breakpoints involved in a complex rearrangement on *SM5* that is associated with the duplication of two genomic regions. Unbiased sequencing of several balancers allowed us to identify stocks with incorrectly identified balancers as well as single and double crossover events that had occurred between *2^nd^* chromosome balancers and their homologs. The confirmed crossover events that we recovered were at least 2 Mb from the closest inversion breakpoint, consistent with observations from other balancer chromosomes. Balancer chromosomes differ from one another both by large tracts of sequence diversity generated by recombination and by small differences, such as single nucleotide polymorphisms (SNPs). Therefore, we also report loss-of-function mutations carried by these chromosomes and unique SNP and InDel polymorphisms present on only single balancers. These findings provide valuable information about the structure of commonly used *2^nd^* chromosome balancers and extend recent work examining the structure of *X* and *3^rd^* chromosome balancers. Finally, these observations provide new insights into how the sequences of individual balancers have diverged over time.

Balancer chromosomes are multiply inverted and rearranged chromosomes that both suppress recombination during meiosis and prevent the recovery of recombinant chromosomes. Balancers in *Drosophila melanogaster* are used for a number of tasks, such as maintaining deleterious alleles in stock, preserving linkage relationships among alleles, and allowing complex stock construction. Most balancers carry recessive lethal or sterile mutations that prevent them from becoming homozygous in stock, as well as dominant visible markers to make their inheritance easy to follow in crosses. Balancers are available for all chromosomes in *D. melanogaster* except the small *4^th^* chromosome, which does not normally undergo exchange, and the *Y* chromosome.

Because balancers were created before DNA sequencing was available, the precise locations of most inversion breakpoints and the nature of most of the marker alleles carried by balancers have remained unknown. Recently, the positions of most of the inversion breakpoints on the *X* chromosome balancer *FM7* and the *3^rd^* chromosome balancers *TM3*, *TM6*, and *TM6B* were reported or confirmed (Miller *et al.* 2016a; b). These analyses revealed that several genes are disrupted by breakpoints including the highly conserved tumor suppressor gene *p53*, which was bisected by an inversion breakpoint on *TM3* (Miller *et al.* 2016a).

Furthermore, whole-genome sequencing (WGS) of multiple *X* and *3^rd^* chromosome balancers revealed that large chromosomal segments had been exchanged with sequences from nonbalancer homologs by meiotic recombination. For example, double crossovers within an inverted segment of the *X* chromosome balancer *FM7c* led to loss of the female sterile allele *sn^X2^* on multiple occasions (Miller *et al.* 2016b). Likewise, double crossovers were reported within inverted segments of the *3^rd^* chromosome balancers *TM3* and *TM6B*, and single-exchange events were found to be common in the unbalanced distalmost 7 Mb of chromosome arm *3L* in *TM3* (Miller *et al.* 2016a). There was also evidence that balancers had diverged at the nucleotide level since their common origins. Here, we present a similar analysis of sequence diversity for the commonly used *2^nd^* chromosome balancers *SM1*, *SM5*, *SM6a*, and *CyO*.

These four *2^nd^* chromosome balancers have their origins in two naturally occurring paracentric inversions of *2L* and *2R* that were on the same chromosome. This chromosome was first described by Ward (1923), who was studying the *Curly* (*Cy^1^*) mutation (recently renamed *Duox^Cy^* (Hurd *et al.* 2015)). She reported that this chromosome (later known as *In(2L)Cy* + *In(2R)Cy*) showed reductions in crossing over in each chromosome arm when heterozygous with normal chromosomes. Sturtevant (1926) proposed that these reductions in exchange were due to paracentric inversions and later (Sturtevant 1931) demonstrated that the gene order had indeed changed for *2L*. A similar study by Graubard (1932) verified the presence of the paracentric inversion on *2R*. By 1936, Calvin Bridges and Ju-Chi Li had confirmed the presence of the two inversions by polytene chromosome analysis and had mapped their breakpoints to 22D;33F for *In(2L)Cy* and 42A;58A for *In(2R)Cy* (cited in Morgan *et al.* 1936; Bridges 1937; and Bridges and Warren 1944). Ward also identified and described a mutation in *cinnabar* (*cn^2^*) on the *In(2L)Cy* + *In(2R)Cy* chromosome.

Although *In(2L)Cy* + *In(2R)Cy* can be used as a *2^nd^* chromosome balancer, it is not very effective because it still allows exchange with a normal sequence homolog, albeit at a reduced frequency (Ward 1923). In an attempt to create a better whole-chromosome balancer, *SM1* was made by irradiating an *In(2L)Cy* + *In(2R)Cy* chromosome marked with *al^2^*, *Duox^Cy^*, *cn^2^*, and *sp^2^* (Lewis and Mislove 1953). Polytene chromosome analysis revealed that irradiation had induced a large pericentric inversion with breakpoints at 22A and 60B. While the addition of an inversion reduced recombination substantially, *SM1* still allows low levels of recombination, especially in the large region of *2R* not disrupted by inversion breakpoints (Lindsley and Zimm 1992). To isolate a still better balancer, Mislove and Lewis (1955) repeatedly X-rayed *SM1*, introducing two additional inversions and a complex rearrangement. This balancer, called *SM5*, is marked with *al^2^*, *Duox^Cy^*, *cn^2^*, *lt^v^*, and *sp^2^*, and is associated with good fertility and viability for a chromosome so extensively rearranged (12 euchromatic breakpoints and, as we will discuss below, at least 4 heterochromatic breakpoints). Unlike the other second chromosome balancers that are euploid, the complex rearrangement in *SM5* resulted in the duplication of segments 42A to 42E and 58A to 58F (Mislove and Lewis 1955).

In 1956, Oster reported a new balancer obtained after irradiating males carrying *In(2L)Cy* + *In(2R)Cy* marked with *Duox^Cy^*, *dpy^lvI^*, *pr^1^*, *cn^2^* (Oster 1956). This balancer is referred to as *CyO* (Lindsley and Zimm 1992). EMS treatment of *CyO* several years after it was created changed the weak allele *cn^2^* to *cn^2P^*, a null allele now present on some, but not all, *CyO* chromosomes (Craymer 1980). Finally, *SM6* was created through a series of single exchanges between *CyO* and *SM1* (Craymer 1984). Two versions of *SM6* were made: *SM6a* marked with *al^2^*, *Duox^Cy^*, *dpy^lvI^*, *cn^2P^*, and *sp^2^*; and *SM6b*, which carries the additional marker *amos^Roi-1^*.

In this study, we report or confirm the genomic positions of a majority of the breakpoints present on *SM1*, *SM5*, *CyO*, and *SM6a*. We also identify two previously unannotated marker mutations carried by these chromosomes and precisely define the two large duplicated segments carried by *SM5*. Furthermore, we find many novel loss-of-function mutations that are both shared and unique among this sample of balancers, demonstrating that, as seen with previous studies of balancers, significant genetic diversity exists among balancer chromosomes derived from single original isolates.

Although significant sequence diversity due to recombination has been observed among different versions of the *X* chromosome balancer *FM7* and the *3^rd^* chromosome balancers *TM3* and *TM6B* (Miller *et al.* 2016a; b), we find that, except for two single exchange events in the distal unbalanced region of *2R* on *CyO* and *SM5*, few large tracts of sequence diversity exist on the *SM5*, *CyO*, or *SM6a* balancers. This suggests that all three of these balancers allow very little recombination with normal-sequence homologs and thus are near-complete balancers for the *2^nd^* chromosome. We do, however, identify several double crossovers within the *SM1* chromosomes that we sequenced, indicating that, as previously reported, it is a poor balancer for a large portion of the right arm of the *2^nd^* chromosome (Lindsley and Zimm 1992). Finally, consistent with previous studies (Miller *et al.* 2016a; b), unbiased sequencing revealed four balancers that had been misidentified in stock genotypes.

## Materials & Methods

### Stocks used for breakpoint identification

All balancers sequenced in this study were obtained from stocks at the Bloomington Drosophila Stock Center (Table S1). Sequence data for four balancers from a previous study (Miller *et al.* 2016a) were also included. Before sequencing, multiple balancer-carrying males were crossed to multiple *ISO-1* virgin females. *ISO-1* is the Drosophila reference genome stock and was obtained in 2014 from the Berkeley Drosophila Genome Project (Hoskins *et al.* 2015).

### DNA preparation and genome alignment

DNA for sequencing was prepared from balancer/*ISO-1* males using the Qiagen DNeasy Blood and Tissue Kit. Flies were starved for 1 hr before freezing at –80°. Libraries were prepared and quantified as described in Miller *et al.* (2016a). All libraries were pooled, requantified, and sequenced in 150-bp paired-end mode on the Illumina NextSeq 500 instrument. Illumina Real Time Analysis version 2.4.6 was run to demultiplex reads and generate FASTQ files following sequencing. Alignment to the *D. melanogaster* reference genome (dm6) was performed using bwa version 0.7.15-r1140 (Li and Durbin 2009). SNPs were called using SAMtools version 1.5 and BCFtools version 1.4.1 (Li *et al.* 2009). DNA preparation, sequencing, and alignment for balancers from stocks 504, 22239, 24759 and the Hawley lab *SM1* stock are described in Miller *et al.* (2016a).

### Identification of balancer breakpoints

Breakpoints were identified as in Miller *et al.* (2016a). Briefly, split and discordant read pairs were isolated using SAMBLASTER (Faust and Hall 2014) from regions where rearrangements were previously reported to be present (Lindsley and Zimm 1992). Split and discordant pairs were then *de novo* assembled and BLAST was used to identify assembled fragments that aligned to two distinct regions of the genome. Breakdancer was used to validate our custom analysis and to search for novel rearrangements (Chen *et al.* 2009).

### PCR and Sanger sequencing

Primers for PCR validation were designed using Primer3 (Rozen and Skaletsky 2000). Six of 10 inversion breakpoints in which the molecular position was confirmed or identified in this study were validated using PCR and Sanger sequencing (Table S2). Briefly, ExTaq polymerase was used according to the manufacturer’s instructions. Extension times and annealing temperature for each breakpoint are given in Table S2.

### Identification of shared and unique SNPs

Shared and unique SNPs were identified using VCFtools version 0.1.15 (Danecek *et al.* 2011). Only SNPs with VCF quality scores >220 were considered for analysis. VCF files were merged using vcf-merge (part of the VCFtools package), which allowed the counting of shared and unique SNPs. SnpEff version 4.3p (Cingolani *et al.* 2012) was used to annotate VCF files. Filtering of annotated VCF files was done using custom scripts.

### Complementation testing

Fly crosses were made on standard medium and reared under routine conditions (details provided on request). Crosses listed in Table S3 evaluated recessive phenotypes associated with balancer breakpoints. Stocks used to evaluate breakpoints were tested with the control crosses listed in Table S4. Genotypes and the sources of stocks are given in Table S1.

### Testis dissection and microscopy

Flies for microscopy were grown on Ward’s Instant *Drosophila* Medium and maintained at 25°. *dpy^ov1^wg^Sp-1^/SM5* males were crossed to *Df(2L)Exel6005/CyO* females and male offspring with genotype *Df(2L)Exel6005/SM5* were selected and used for testis dissections. *dpy^ov1^wg^Sp-1^/Df(2L)Exel6005* males were used as a control. Testes were dissected in TB1 buffer (15 mM 1:1 K_2_HPO_4_:KH_2_PO_4_, pH 6.7, 80 mM KCl, 16 mM NaCl, 5 mM MgCl_2_, 1% PEG 6000) on a microscope slide. A coverslip was placed on the dissection and excess buffer was drained slowly using a Kimwipe. Samples were visualized and documented using the phase-contrast setting of an Olympus BX60 Upright Compound Microscope equipped with an Olympus DP73 Color Camera.

### Data availability

All stocks are available from the Bloomington Drosophila Stock Center, with the exception of the Hawley lab *SM1*; *TM3* stock, which is available upon request. Raw sequencing data for all samples have been uploaded to the National Center for Biotechnology Information (NCBI) at http://ncbi.nlm.nih.gov and can be found under BioProject PRJNA413446. Sequencing data for balancers from stocks 504, 22239, 24759 and the Hawley lab *SM1* stock were submitted to NCBI previously and can be found under BioProject PRJNA315473. Scripts used to align data, call SNPs, create heatmaps, and identify shared and unique mutations are available on Github: https://github.com/danrdanny/2ndChromosomePaper. Original data underlying this manuscript can be accessed from the Stowers Original Data Repository at http://www.stowers.org/research/publications/libpb-1257.

## Results

### Sequencing and identification of inversion breakpoints

To identify the breakpoints carried by the *2^nd^* chromosome balancers *SM1*, *SM5*, *CyO*, and *SM6a*, we crossed 18 stocks carrying one of these balancers to the Drosophila reference genome stock, *ISO-1*, and selected balancer/*ISO-1* males for WGS. We also analyzed data from four *2^nd^* chromosome balancers sequenced previously (Miller *et al.* 2016a) (Table 1). Among this panel of 22 balancer chromosomes, we identified four cases where balancers had been incorrectly identified in the original stocks (Table 1). Throughout the text we refer to the mislabeled stocks by their stock numbers and group them with their correct balancer chromosome in figures.

**Table 1:** Balancer stocks used in this study

**Stock number*^1^***	**Genotype**	**Label**	**Actual**
223	*ap^4^/SM5*	*SM5*	—
240	*nw^B^/SM5*	*SM5*	—
325	*l(2)39a^1^ px^1^ slt^1^ sp^1^/SM5*	*SM5*	*SM1*
400	*sm^1^ px^1^ pd^1^/SM5*	*SM5*	—
405	*dpy^ov1^ wg^Sp-1^/SM5*	*SM5*	—
1143	*Df(2R)en28/SM5*	*SM5*	—
1465	*Df(2R)Dll-MP/SM6a*	*SM6a*	—
6853	*y^1^ w^67c23^*; *Df(2R)01D01W-L053/SM6a*	*SM6a*	—
8785	y^1^ w*; *sax^5^/SM6a*	*SM6a*	*CyO*
9162	*Rca1^IX^ cn^1^ bw^1^/SM6a*	*SM6a*	—
23663	*w^1118^*; *Df(2L)BSC278/SM6a*	*SM6a*	—
24380	*w^1118^*; *Df(2R)BSC356/SM6a*	*SM6a*	—
Hawley lab stock***^1^***	*wg^Sp-1^/SM6a*; *Pri^1^ Dr^1^/TM3*, *Sb^1^ Ser^1^*	*SM6a*	*SM1*
2	w*; *betaTub60D^2^ Kr^If-1^/CyO*	*CyO*	—
31	*w^118^*; *Df(2R)H3D3/CyO*	*CyO*	—
471	*Bkd^M^/CyO*	*CyO*	—
504***^1^***	*amd^21^ Bl^1^/CyO*; *DCTN1-p150^1^/TM3*, *Sb^1^ Ser^1^*	*CyO*	—
533	*stil^3^/CyO*	*CyO*	—
1602	*Df(2L)TW65/CyO*	*CyO*	—
3076	*Df(2L)E55*, *rdo^1^ hook^1^ Lar^E55^ pr^1^/CyO*	*CyO*	—
22239***^1^***	y^1^ w*; *P{y^+t7.7^ = Mae-UAS.6.11}Dpit47^LA00491^/CyO*; l(3)**/TM3, *Sb^1^ Ser^1^*	*CyO*	—
24759***^1^***	w*; *sna^Sco^/CyO*; *P{w^+mC^ = ninaD-GAL4.W}3/TM3*, *Sb^1^*	*CyO*	*SM1*

1 *2^nd^* chromosome balancers sequenced as part of Miller *et al.* 2016a are indicated after their stock number.

**Figure 1:**
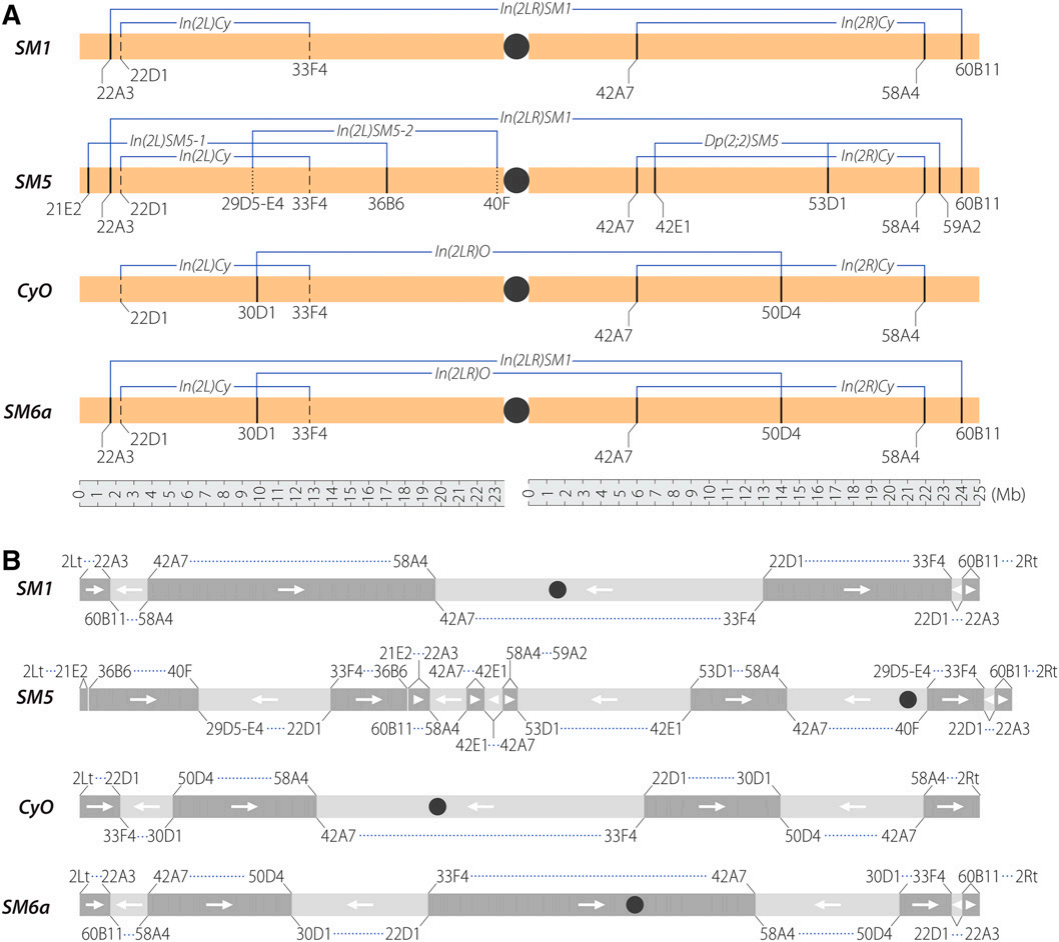
Second chromosome balancer inversion breakpoints and rearrangements. Chromosome bands here and in the text are those predicted from the genomic coordinates of breakpoints in Table 2. (A) Breakpoints whose genomic positions are known are shown as solid lines, those approximated (22D1 and 33F4) are shown as dashed lines, and those with unknown genomic coordinates (29D5–E4 and 40F) are shown as dotted lines. (B) Multiple rearrangements and inversions have resulted in novel configurations for each second chromosome balancer.

Previous studies based on polytene squashes reported the presence of 14 euchromatic breakpoints and one heterochromatic breakpoint on the *SM1*, *SM5*, *CyO*, and *SM6a* balancer chromosomes (Table 2; Figure 1) (summarized in Lindsley and Zimm 1992). Eleven of the euchromatic breakpoints as well as the heterochromatic breakpoint are associated with simple inversions. The remaining three euchromatic breakpoints are associated with the complex rearrangement on *SM5* that duplicated two genomic regions: 42A7–42E1 and 58A4–59A2 (Table 2).

**Table 2:** Breakpoints on *2^nd^* chromosome balancers

**Balancer(s)**	**Component aberration**	**Polytene breakpoint*^1^***	**Proximal breakpoint coordinate**	**Distal breakpoint coordinate**	**Duplication (+) or deletion (–)**	**Predicted band*^2^***	**Disrupts**
*SM1*, *SM5*, *SM6a*, *CyO*	*In(2L)Cy*	22D1–2	Unknown*^3^*	Unknown*^3^*	Unknown	22D1	*CG11723*, *TBCD*, or *AIF^4^*
		33F5–34A1	Unknown*^5^*	Unknown*^5^*	Unknown	33F4	*MRP*
*SM1*, *SM5*, *SM6a*, *CyO*	*In(2R)Cy*	42A2–3	2R:6,012,459	2R:6,012,739	+280	42A7	5′ of *Src42A*
		58A4–58B1	2R:21,971,918	2R:21,972,072	−153	58A4	
*SM1*, *SM5*, *SM6a*	*In(2LR)SM1*	22A3–22B1	2L:1,586,845	2L:1,586,840	−4	22A3	*haf*, *CG10869*
		60B–60C	2R:24,117,046	2R:24,117,059	−12	60B11	*CG3257*
*SM6a*, *CyO*	*In(2LR)O*	30E–30F	2L:9,805,575	2L:9,805,567	−7	30D1	*nAChRα6*
		50C10–50D1	2R:14,067,771	2R:14,067,782	−10	50D4	*Prosap*
*SM5*	*In(2L)SM5-1^6^*	21D1–2	2L:675,187	2L:675,190	+4	21E2	*ds*
		36C	2L:16,995,337	2L:16,995,336	+2	36B6	
*SM5*	*In(2L)SM5-2^6^*	29C–29E	Unknown*^7^*	Unknown*^7^*	Unknown	29D5–E4*^8^*	Unknown
		40F	Unknown	Unknown	Unknown	Unknown	Unknown
*SM5*	*Dp(2;2)SM5^6^*	42D	Unknown*^9^*	6,917,406	Unknown	42E1	*CG30158^10^*
		53C	2R:16,682,351	2R:16,682,827	−475	53D1	*CG30463*
		58F	2R:22,689,962	2R:22,689,962	0	59A2	*CR44763^11^*

1 Breakpoint observed in polytene chromosome preparations (Lindsley and Zimm 1992).

2 Breakpoint position predicted from genomic coordinate using FlyBase correlation table.

3 Breakpoint mapped to the interval 2L:2,146,403–2,156,403.

4 One or none of these three genes may be affected by the breakpoint.

5 Breakpoint mapped to the interval 2L:12,726,221–12,736,221.

6 Because *SM5* arose from *SM1* through two inversions (*In(2L)SM5-1* followed by *In(2L)SM5-2*) and a complex rearrangement, we have given symbols to these component aberrations to replace the single aberration *In(2LR)SM5*. The symbol *Dp(2;2)SM5* was chosen for the complex rearrangement to emphasize the duplicated segment from the progenitor over the inverted segment.

7 This breakpoint could not be localized molecularly, but recessive lethality presumably associated with the breakpoint was mapped to the interval 2L:8,529,124–8,700,124 by complementation tests with molecularly defined chromosomal deletions.

8 Bands corresponding to the 2L:8,529,124–8,700,124 interval defined by chromosomal deletions.

9 The proximal side of the breakpoint is present in two presumably identical copies juxtaposed to low-complexity sequence and maps to the 2L:6,916,809–6,917,405 interval.

10 The sequence on the distal side of the breakpoint suggests this gene is disrupted.

11 A second, intact copy of this gene is present elsewhere on *SM5*.

Using a combination of standard and large-insert libraries, we confirmed or identified the precise molecular positions of 8 of the 11 euchromatic inversion breakpoints (Table 2) and validated 6 of those using PCR and Sanger sequencing (Table S2). Although we were unable to identify the precise positions of three of these breakpoints (Table 2), we were able to map the two *In(2L)Cy* breakpoints in polytene bands 22D1 and 33F4 to within 10 kb using large-insert libraries. The exact position of the euchromatic 29D5–E4 breakpoint on *SM5* was difficult to determine because the 40F breakpoint of the 29D5–E4 to 40F inversion lies in centric heterochromatin, making it challenging to analyze using either short-read or large-insert libraries. Nevertheless, we will present evidence below that it can be localized to the 16-gene interval *2L*:8529124–8700124 corresponding to 29D5E4.

**Figure 2:**
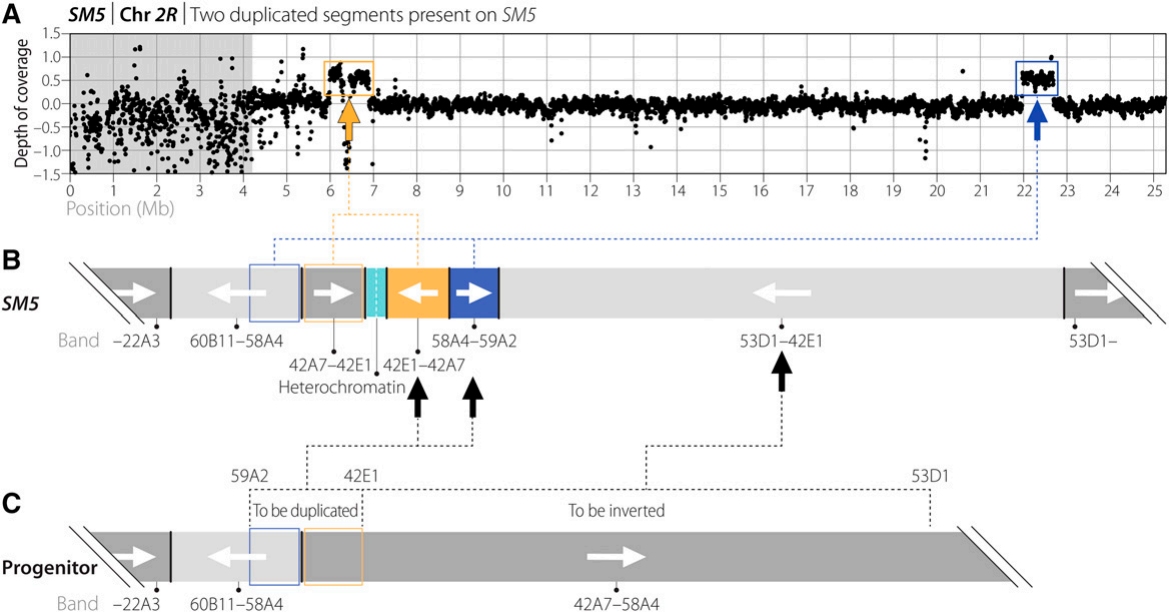
*SM5* carries a complex rearrangement that duplicates two chromosomal segments. (A) Depth-of-coverage analysis of *SM5 2R* indicates that two regions on the standard map are duplicated (orange and blue boxes). The gray shaded area represents centric heterochromatin, which appears heterogeneous due to the difficulty of aligning short-read data to repetitive sequences. (B) The two duplicated segments lie adjacent to one another on *SM5*. One segment (highlighted in orange) duplicates 42A7–42E1 while the other (highlighted in blue) duplicates 58A4–59A2. Note the mirror-image arrangement of the two segments present in two copies with heterochromatin (turquoise) separating them. The two duplicated segments are joined to the end of the 53D1–42E1 segment, which is inverted relative to the progenitor. (C) The 58A4;42A7 inversion breakpoint is present within the duplication on *SM5* because it was present on the progenitor chromosome in the region that was duplicated (denoted by blue and orange boxes). The 42E1–53D1 interval was inverted as part of the complex rearrangement that is now present on *SM5*.

The complex rearrangement on *SM5* (*Dp(2;2)SM5*) that duplicated 42A7–42E1 and 58A4–59A2 is shown in Figure 2. From the order and orientations of chromosomal segments in *SM5*, it is apparent this rearrangement was induced on an *SM1* chromosome that already had two additional inversions (*In(2L)SM5-1* and *In(2L)SM5-2*) (Table 2, Figure 2C). Euchromatic breaks in 42E1, 53D1 and 59A2 were involved to invert the 42E1–53D1 segment and insert a second copy of a segment from the progenitor chromosome that spanned an existing inversion breakpoint and comprised two subsegments (42E1–42A7 and 52A4–59A2) (Figure 2B) (Mislove and Lewis 1955; Lindsley and Zimm 1992). Our sequencing showed the presence of low-complexity sequences juxtaposed to the distal ends of both 42A7–42E1 segments, and they provide a clue to how the unusual mirror-image arrangement of segments may have arisen. They suggest the distal end of the 42A7–42E1 segment in the progenitor was first joined to a heterochromatic region and then, after replication, breaks within the heterochromatic region on sister chromatids joined to produce the mirrored configuration. The exact overall configuration of the intermediate rearrangement is not clear, but it likely involved inversion of the 42E1–53D1 segment as well. The low-complexity sequences made it difficult to determine the exact distal extent of the 42A7 to 42E1 segments (Table 2), and to know if there are sequences deleted or duplicated relative to the distal side of the 42E1 breakpoint. We determined the minimal extent of the duplicated 42A7 to 42E1 segment to be *2R*:6,012,459–6,916,809 (Table 3; Figure 2A), an interval containing 83 protein-coding genes, 14 non-protein-coding (CR) genes, and 19 tRNA genes (r6.17 annotations) (Table S5). In contrast, it was relatively straightforward to characterize the 42E1;53D1 and 53D1;59A2 junctions, which flank the inverted segment and join it to the duplicated segment. Depth of coverage analysis allowed us to determine the precise extent of the duplicated 58A4 to 59A2 segment to be *2R*:21,972,072–22,689,962 (Table 3, Figure 2A), an interval containing 117 protein-coding genes, 18 CR genes, and 6 snoRNA genes (Table S5). Knowing exactly which genes are duplicated is valuable for the construction of stocks. For example, *SM5* has been used to maintain deficiencies of the haploinsufficient locus *M(2)58F*, which corresponds to *RpS24* and/or *RpS16*, two genes contained within the 58A4 to 59A2 duplicated segment (Marygold *et al.* 2007).

**Table 3:** Details of the duplicated segments on *SM5*

**Balancer**	**Start**	**End**	**Size**	**Predicted bands*^1^***
***SM5***	2R:6,012,459	2R:6,916,809*^2^*	904,350	42A7–42E1
	2R:21,972,072	2R:22,689,962	712,929	58A4–59A2

1 Breakpoint position predicted from genomic coordinates using FlyBase correlation table.

2 This coordinate represents the minimal distal extent of this interval. The maximal extent is 2R:6,917,405.

### Inversion breakpoints affect protein-coding genes

Several of the breakpoints we identified or confirmed lie within the transcribed regions of protein-coding genes (Table 2). Breakpoints that lie in intergenic regions and those we mapped to small intervals may also affect the activities of genes. To test the breakpoints for strong phenotypic effects, we performed complementation tests with deficiencies spanning these breakpoints and scored for lethality, female sterility, or grossly abnormal morphology (Table S6). Only two of the breakpoints, 21E2 and 29D5–E4 on *SM5*, gave phenotypes in these tests. The 21E2 breakpoint in the gene *dachsous* (*ds*) was lethal, with the few escapers having the short appendages typical of *ds* mutants, consistent with previous reports (Craymer 1980; Clark *et al.* 1995). The 29D5–E4 breakpoint on *SM5* also appears to be lethal. Four deletions chosen to span the region of the breakpoint defined by polytene chromosome analysis (29C–E; (Lindsley and Zimm 1992)) were all lethal in combination with *SM5*. Assuming that the inversion breakpoint is the only lethal mutation present, it maps to the 16-gene region common to all the deletions (*2L*:12,726,221–12,736,221), which corresponds to 29D5–E4. While the 30D1 breakpoint disrupting *nAChRα6* on *SM6a* and *CyO* is not associated with lethality or female sterility, previous studies showed that it and other loss-of-function *nAChRα6* mutations confer insecticide resistance (Perry *et al.* 2007; Watson *et al.* 2010).

We were unable to evaluate the phenotypic effects of two breakpoints. The 59A2 breakpoint of the complex rearrangement on *SM5* disrupts *CR44763*, but an intact copy is present elsewhere on *SM5*. The 42E1 breakpoint of the complex rearrangement appears to disrupt *CG30158*, because the sequence of the 42E1–53D1 junction shows that the break lies at 2R:6,917,406 within an intron. Nevertheless, the difficulties we encountered in characterizing the 42E1 ends of the mirror-image 42A7–42E1 segments leave us unable to say with certainty that they terminate at the same site. If, as we expect, the break is not unusually complicated and *CG30158* is disrupted, our complementation tests (Table S6) indicate that knocking out *CG30158* has no severe consequences.

**Figure 3:**
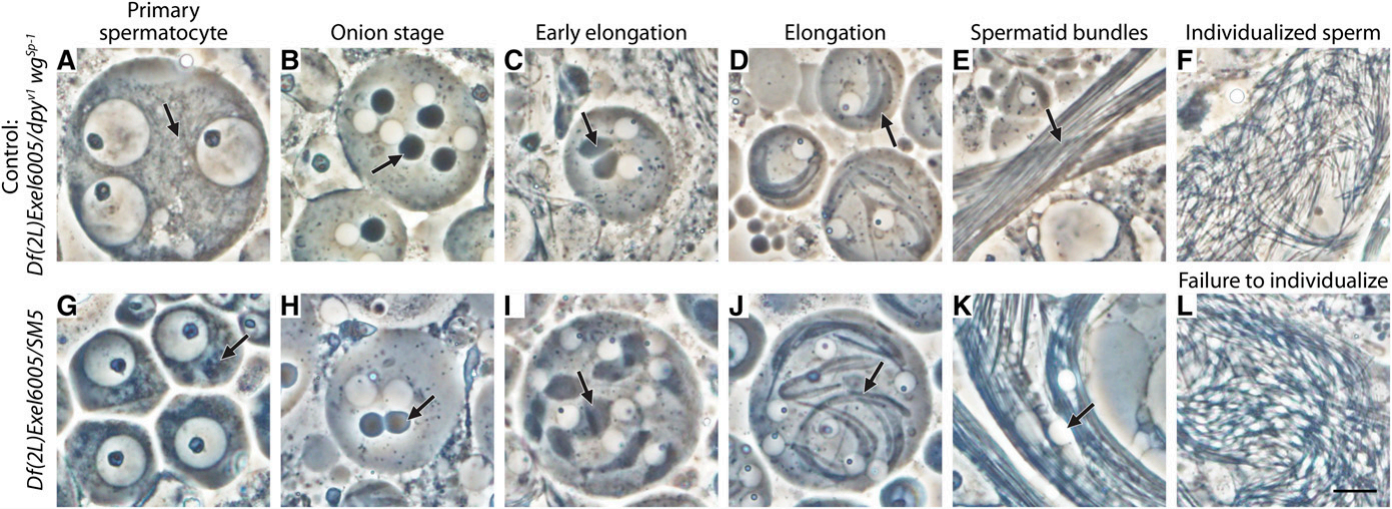
Spermatogenesis phenotypes from disrupting *CG10869*. The noncomplementation of *Df(2L)Exel6005* and the *SM5* inversion breakpoint in *CG10869* results in recessive male sterility and degradation of late elongation-stage spermatid bundles. Phase-contrast micrographs of live squashed testis preparations from (A–F) *Df(2L)Exel6005/dpy^ov1^ wg^Sp-1^* (indistinguishable from wild type) and (G–L) *Df(2L)Exel6005/SM5* males. (A, G) Mitochondria are phase dark, small, and diffuse throughout the cytoplasm in primary spermatocytes (arrows denote mitochondria). (B, H) After meiosis, mitochondria aggregate and fuse beside each phase light nucleus, forming the nebenkern (arrows denote nebenkern), which appears normal in testes from *Df(2L)Exel6005/SM5* males. (C, D, I, J) The nebenkern unfurls, and mitochondrial derivatives appear to elongate normally beside the growing flagellar axoneme during early elongation stages (arrows denote elongating mitochondrial derivatives). Cysts of 64 spermatids elongate together in smooth bundles (E) and later individualize (F) in the control, while *Df(2L)Exel6005/SM5* spermatid bundles (K) appear vacuolated (arrow), suggesting tissue degradation, and sperm fail to individualize (L). The syncytial appearance of cells in many panels is a well-known artifact of live testis squash preparations in which ring canals between cells in a cyst are often broken open. Scale bar, 10 µm.

Although it did not confer lethality or female sterility in complementation tests, the 22A3 breakpoint on *SM1*, *SM5*, and *SM6a* bisects the gene *CG10869*, which is expressed only in adult testes (Chintapalli *et al.* 2007). Complementation testing of *SM5* with *Df(2L)Exel6005*, which encompasses *CG10869*, showed that males were sterile and that sperm failed to individualize during spermatogenesis (Figure 3). Consequently, we have renamed this gene *no individualized sperm* (*nis*). Although *nis* lies within an intron of *hattifattener* (*haf*), it is unlikely that disruption of *haf* contributes to male sterility, because *haf* shows negligible expression in the testis (Chintapalli *et al.* 2007). We did not examine *SM5/Df(2L)Exel6005* flies for the incompletely penetrant muscle innervation defects seen when *haf* expression is reduced by RNAi or mutations (Kurusu *et al.* 2008).

That we were able to discover a previously unknown mutation and determine the role of an uncharacterized gene demonstrates the value of molecularly mapping the inversion breakpoints on these commonly used balancer chromosomes. Furthermore, knowledge of the genes disrupted by inversion breakpoints is useful for researchers studying those genes in terms of their choice of balancer.

### Sequence diversity among 2^nd^ chromosome balancers

**Figure 4:**
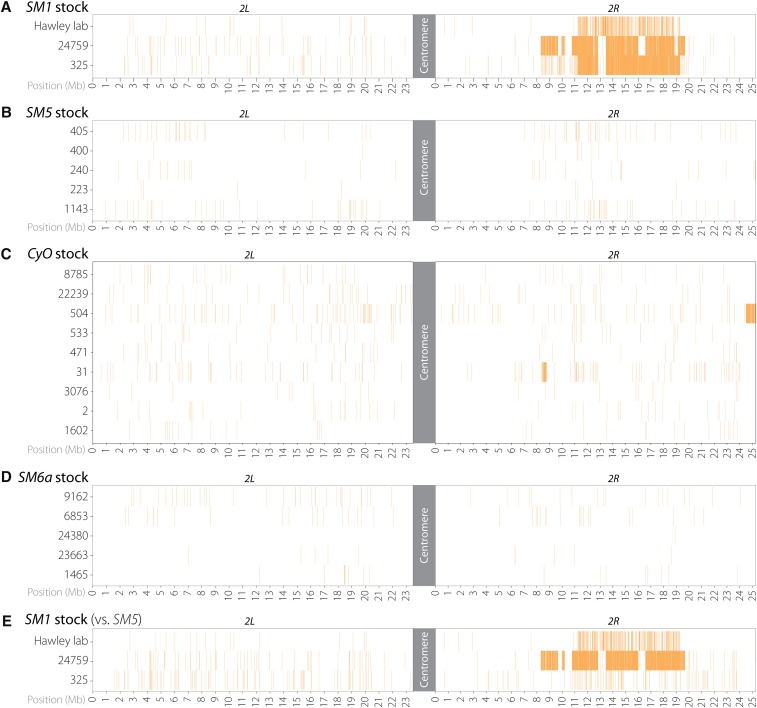
Heatmaps of unique SNPs reveals sequence diversity among *2^nd^* chromosome balancers. (A) Extensive sequence diversity appears to exist on the right arm of the *2^nd^* chromosome among *SM1* balancers from three stocks, indicating that these regions are susceptible to double crossover events. (B–D) Little sequence diversity exists among the *SM5*, *CyO*, and *SM6a* chromosomes sequenced, except for a single exchange event on the distal *2R* tip of *CyO* from stock 504. One apparent double crossover event on *CyO* from stock 31 is actually a duplication. (E) Comparing SNPs present on all *SM1* stocks to *SM5*, which *SM5* carries the ancestral *SM1* polymorphism profile, reveals that *SM1* chromosomes from two stocks experienced double crossover events while *SM1* from stock 325 did not.

**Figure 5:**
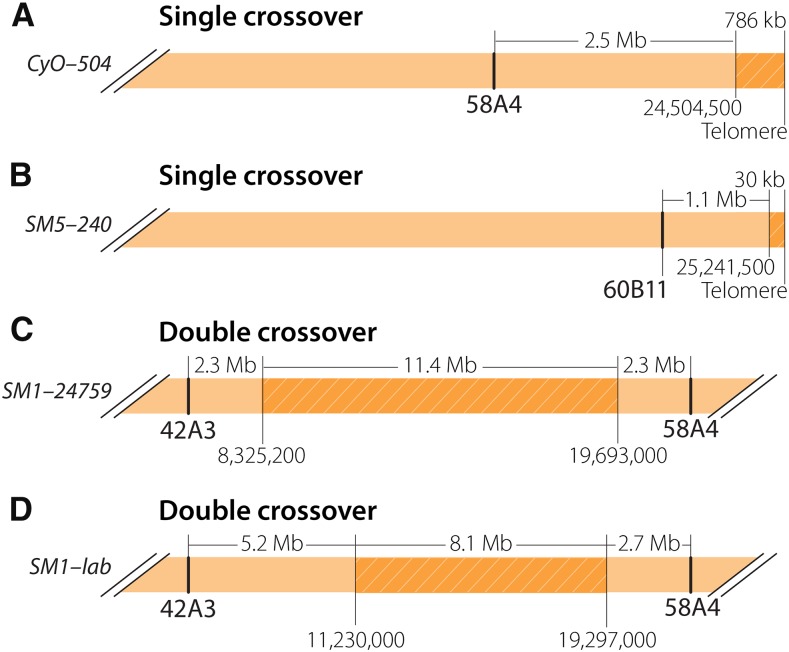
Single (SCO) and double (DCO) crossover events recovered in this study. (A) The SCO at the distal tip of *CyO* from stock 504 lies 2.5 Mb distal to the distalmost breakpoint at 58A4. (B) An SCO occurred approximately 30 kb from the *2R* telomeric repeats on *SM5* from stock 240. It is unclear whether this SCO was the result of a meiotic crossover or another kind of exchange. (C-D) The DCO exchanges in the *SM1* chromosomes from stocks 24759 and the Hawley lab stock lie at least 2.3 Mb from the inversion breakpoints flanking the DCO.

As seen in studies of *X* and *3^rd^* chromosome balancers (Miller *et al.* 2016a; b), stretches of unique SNPs indicate the occurrence of recombination events between balancers and nonbalancer homologs. To investigate how much sequence diversity, if any, exists among each of the four *2^nd^* chromosome balancers, we plotted the positions of SNPs that were unique among balancers of the same type and found very few large tracts on the *SM5*, *CyO*, and *SM6a* balancers (Figure 4 B-D). Two exchange events were observed at the distal tip of *2R*: one on *CyO* from stock 504 (Figure 4C, Figure 5A) and one on *SM5* from stock 240 (Figure 5B). The single crossover (SCO) on *CyO* occurred at approximately *2R*:24,509,500, ∼2.5 Mb away from the distalmost 58A4 breakpoint. The SCO on *SM5* occurred at approximately *2R:25,241,500*, ∼1.1 Mb away from the distalmost 60B11 inversion breakpoint. What appears to be a double crossover (DCO) event on *CyO* from stock 31 (Figure 4C) is in reality a duplication event discussed below.

Unlike the other three balancers, we did find extensive sequence diversity within one region of *2R* among the three *SM1* chromosomes sequenced (Figure 4A). This was not surprising, as it was previously noted that the large 16-Mb interval between the 42A7 and 58A4 inversion breakpoints is vulnerable to DCO events (Lindsley and Zimm 1992). It is possible that one of the three *SM1* sequences is identical to the sequence of the original *SM1* balancer and the other two sequences represent DCO events, or that all three sequences represent DCO events. To determine which scenario is correct, we compared the SNP distribution of each *SM1* chromosome against *SM5*, which was created from *SM1*, and which we assume is an ancestral snapshot of *SM1*. This revealed that only the *SM1* chromosomes from stock 24759 and the Hawley lab stock had experienced DCO events (Figure 4E, Figure 5C-D). That only two of the three *SM1* chromosomes differed from *SM5* suggests that the *SM1* chromosome used to make *SM5* carried the SNP profile of the original *SM1* chromosome.

Interestingly, the crossovers giving rise to these DCOs on *SM1* occurred at least 2.3 Mb away from the flanking inversion breakpoints (Figure 5C-D). This distance and the ∼2.5 Mb distance observed for the SCO on *CyO* from stock 504 are consistent with previous observations that found SCO events between the *3^rd^* chromosome balancer *TM3* and a normal-sequence chromosome occurred no closer than ∼2 Mb to an inversion breakpoint (Miller *et al.* 2016a; b). The SCO that occurred ∼1.1 Mb from the distalmost breakpoint on *2R* in *SM5* from stock 240 is challenging to interpret in this regard. It lies approximately 30 kb from telomeric repeats, and a region with increased recombination was reported previously for a similar subtelomeric region of the *X* chromosome (Anderson *et al.* 2008). This SCO may reflect a similar region of increased recombination on *2R*. Whether subtelomeric exchanges reflect normal meiotic recombination or events associated with the maintenance of chromosome ends remains unclear (Kern and Begun 2008).

### Structural differences exist among balancer stocks

**Figure 6:**
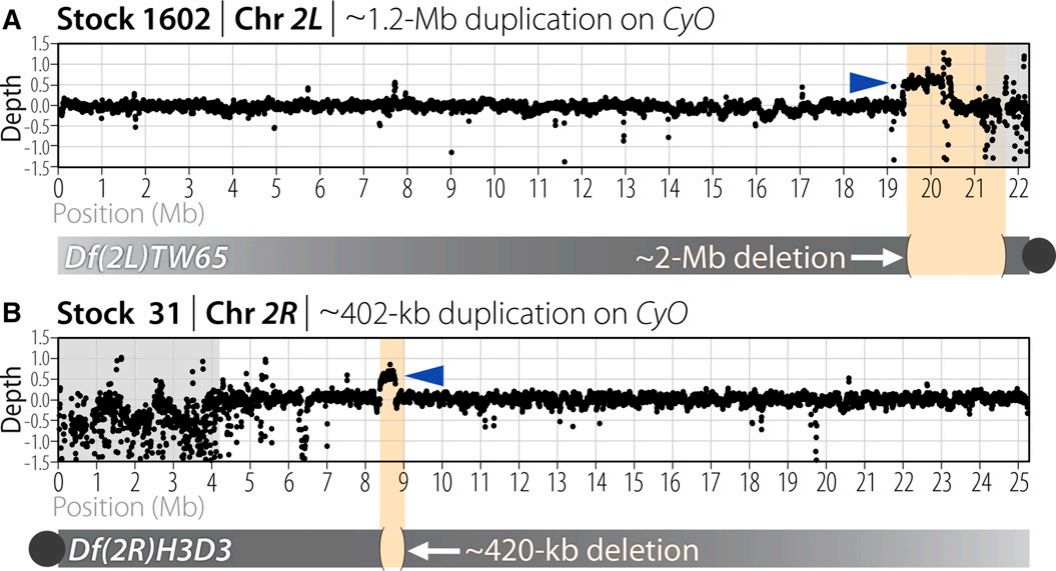
Duplications present in stocks where a deficiency was maintained with a *2^nd^* chromosome balancer. (A) *CyO* from stock 1602 carries a 1.2-Mb tandem duplication (blue arrow) that partially covers the *Df(2L)TW65* deficiency (orange shading). (B) Stock 31 had a 402-kb duplication (blue arrow) that may have been present on *CyO*. The SNPs present within this duplicated segment indicate that it did not come from the *CyO* chromosome itself, but from an unknown second chromosome. The gray shaded areas represent centric heterochromatin.

Unbiased sequencing of a large number of *2^nd^* chromosome balancers also allowed us to identify novel and unexpected structural events within each stock. For example, we sequenced six balancers from stocks with deficiencies (Table 1) and identified two novel duplications (Figure 6). The *CyO* in stock 1602 balances *Df(2L)TW65*, a deletion of 37F–39E (approximate coordinates *2L*:19,675,000–21,700,000), and carries a novel 1.2-Mb tandem duplication with coordinates *2L*:20,422,204–21,606,670. Stock 31 contained *CyO* balancing *Df(2R)H3D3*, a deletion of 44D–44F (approximate coordinates *2R*:8,430,000–8,850,000), and it carried a novel 402-kb duplication with approximate coordinates *2R*:8,376,000–8,777,700. This duplicated region carried polymorphisms not seen on any other *CyO* balancer, suggesting that it originated from a chromosome other than *CyO* (Figure 4C). Both its proximal and distal ends were bounded by low-complexity or repetitive sequence, making it difficult to determine using paired sequencing reads where it was positioned in the genome. Because the sequencing depth of the duplicated segment was 50% higher than background, the duplicated segment appears to have co-segregated with *CyO* in the outcross to *ISO-1*. Unfortunately, the *Df(2R)H3D3* stock was rebalanced at the Bloomington Stock Center shortly after the sequence analysis, so follow-up mapping to prove *CyO* carried the duplication was impossible. Regardless, these observations show that there are unique structural variations within balancer stocks and that balancers themselves can carry duplications. Neither of these duplications contain any of the haploinsufficient genes cataloged by Cook *et al.* (2012), yet they both partially overlap corresponding deficiencies and restore many genes to their normal two copies, which likely explains why they were retained and why, in general, a balancer carrying a duplication can have a competitive advantage when it arises in a stock.

### Marker alleles carried by 2^nd^ chromosome balancers

Whole-genome sequencing provides an opportunity to identify and define the molecular nature of marker alleles carried by balancer chromosomes. Among these four balancers, there are both shared and unique visible mutations, which may be used to differentiate the balancers. For example, *SM1* is marked with *al^2^ Duox^Cy^ cn^2^ sp^2^*, while *SM5* carries those markers as well as *ds^55^* and *lt^v^*, and *SM6a* is marked with *al^2^ Duox^Cy^ dpy^lvI^ cn^2P^ sp^2^*. Most *CyO* balancers, meanwhile, are marked with *Duox^Cy^ dpy^lvI^ pr^1^ cn^2^*, although some have *cn^2P^* instead of *cn^2^* (Craymer 1980). Of these nine marker alleles, five (*ds^55^*, *Duox^Cy^*, *pr^1^*, *cn^2^*, and *sp^2^*) have been sequenced previously, and we were able to confirm the molecular nature of all five (Table 4) as well as the nature of the *nAChRα6°* mutation associated with insecticide resistance (Perry *et al.* 2007; Watson *et al.* 2010). We also identified molecular abnormalities in two (*al^2^* and *lt^v^*) of the four previously unsequenced markers (*al^2^*, *dpy^lvI^*, *lt^v^*, *and cn^2P^*). Note, however, that *lt^v^* is a variegating allele of *lt*, and it is possible that the 40F breakpoint in *SM5* induces position-effect variegation rather than the variegation being attributable to the transposon insertion we identified. We were unable to determine the nature of the lesions in *dpy^lvI^* and *cn^2P^*.

**Table 4:** Marker alleles present on *2^nd^* chromosome balancers

**Balancers**	**Allele**	**Mutation**
*SM1*, *SM5*, *SM6a*, *CyO*	*Duox^Cy^*	Nonsynonymous C-to-A mutation changing Gly 1505 to Cys, consistent with Hurd *et al.* (2015).
*SM1*, *SM5*, *CyO*	*cn^2^*	*cn^2^* has two *roo* insertions: one with a 70-nt target site duplication (TSD) of 2R:7,784,487–7,784,556 that includes the 1^st^ intron and 2^nd^ exon of *cn*, and the other in the 1^st^ intron of *cn* with a 6-nt TSD of 2R:7,784,682-7,784,687. Previously reported as an 8-kb insertion in *cn* (Warren *et al.* 1996).
*SM6a*, *CyO*	*cn^2P^*	*cn^2P^* was induced by EMS treatment (Craymer 1980), and is present on some *CyO* chromosomes and all *SM6a* and *SM6b* chromosomes. Using our dataset, we are unable to identify the difference between *cn^2^* and *cn^2P^*.
*SM1*, *SM5*, *SM6a*	*al^2^*	Previously unknown. 10-nt deletion in 4^th^ exon of *al* resulting in a frameshift. Deletion is from 2L:386,781–386,793. The deletion may be a single deletion or two deletions, as 3 nt of sequence aligns in multiple positions within the 13-nt interval.
*SM1*, *SM5*, *SM6a*	*sp^2^*	*412* insertion in 5′ UTR of *Dat*. Previously known (Eric Spana, personal communication).
*CyO*	*pr^1^*	Previously reported *412* insertion at 2L:20,074,872 (Kim *et al.* 1996).
*SM5*	*ds^55^*	21D2–36C inversion breakpoint is in 1^st^ intron of *ds*. Previously reported (Clark *et al.* 1995).
*SM5*	*lt^v^*	Previously unknown. *Doc* insertion at/near 2L:22,927,436, which may disrupt the splice acceptor site of the 6^th^ exon. Alternatively, the variegating phenotype may be due to the 40F breakpoint, which may be near *lt*.
*CyO*	*nAChRα6°*	30F–50D inversion breakpoint is within an intron of *nAChRα6*. Previously reported (Perry *et al.* 2007).
*CyO*	*dpy^lvI^*	Unknown.

### Mutations that are shared by and unique among balancer chromosomes

Many balancer chromosomes were created using X-ray mutagenesis, which generated not only useful inversions but also unrecognized mutations. Furthermore, as balancers have been kept in stock, they have diverged from one another over time through the accumulation of *de novo* SNP and InDel polymorphisms. Finally, *cn^2P^* was induced by EMS treatment, which undoubtedly increased the number of mutations present on balancers carrying this marker (Craymer 1980).

From the five *SM5*, nine *CyO*, and five *SM6a* balancers sequenced, we identified 234,623 high-quality SNPs shared by at least two of these three balancer types. (We did not include *SM1* in this analysis because we felt that three stocks, two of which had experienced large DCO events, were not sufficient to determine accurately which mutations were shared or novel.) We used SnpEff (Cingolani *et al.* 2012) to determine how many of these SNPs affect genes (both protein-coding and noncoding) and found 35 nonsense mutations, 62 splice site mutations, 9 start-loss mutations, and 8,898 missense mutations (summary statistics in Table 5, list of all shared mutations in Table S7). We also found 1,558 high-quality SNPs shared only among all *SM5* balancers, 13,888 shared only among all *CyO* balancers, and 20,567 shared only among all *SM6a* balancers. These polymorphisms also introduce nonsense, splice acceptor, and missense mutations that are unique to these chromosomes (Table 5, Table S7). For example, all *CyO* balancers we sequenced have nonsense mutations in 2 genes, *CG33310* and *CG31750*, not present on any of the *SM5* or *SM6a* balancers sequenced. Finally, we identified an average of 2,627 unique SNPs per individual balancer chromosome. These unique SNPs resulted in a total of 683 unique missense mutations, 10 unique splice site mutations, and 11 unique nonsense mutations among all the balancers we studied (Table 5, list of all unique mutations in Table S8). As an example, *CyO* from stock 1602 has a nonsense mutation in *Nplp4* that is not observed on any other balancer. That there are many mutations affecting genes both shared and unique among balancer chromosomes demonstrates that stock-to-stock variability exists among balancers and this variability may impact the fitness of stocks or the interpretation of experimental results.

**Table 5:** Shared or unique mutations carried by *2^nd^* chromosome balancers

	**Mutation type**			
**Balancer**	**Missense**	**Splice site**	**Nonsense**	**Start-loss**
All *SM5* only	49	0	1	0
All *CyO* only	548	2	2	0
All *SM6a* only	520	0	0	0
Shared among all balancers	8898	62	35	9
Present on only one balancer	683	10	11	0

## Discussion

Whole-genome sequencing of balancer chromosomes provides several important types of information. For example, it can tell us whether a balancer carries any mutations aside from its marker alleles, whether it has acquired any additional structural variations such as duplications or deletions, and which of its regions have undergone crossing over. It can also help us determine the exact molecular locations of breakpoints, which are useful for understanding how breakpoints may directly or indirectly affect genes.

Knowledge of the exact positions of inversion breakpoints has important consequences for choosing the appropriate balancer for maintaining an allele in stock. Because these breakpoints often bisect or lie very close to genes, they can disrupt gene function or affect it by position-effect suppression. For example, the 15D3 inversion breakpoint on the *FM7* balancer bisects the predicted peptidase gene *CG45002* (Miller *et al.* 2016b), and the 84B1 inversion breakpoint on *TM6B* affects the regulatory region of *Antp* (Miller *et al.* 2016a). In this study, we report or confirm the exact or approximate positions of eleven euchromatic inversion breakpoints present on the *2^nd^* chromosome balancers *SM1*, *SM5*, *CyO*, and *SM6a* (Table 2) along with the positions of the three euchromatic breakpoints of the duplication-associated complex rearrangement on *SM5* (Table 3). Knowing the exact genes carried by the duplicated segments is important for researchers studying genes in these intervals who may not realize that their gene of interest is present in three copies.

Moreover, we find that balancers are not immune to *de novo* structural variation that may provide selective advantages in stock. In our study, two out of six balancers maintained in stock over a deficiency were associated with a duplication covering a large portion of the deficiency (Figure 6). At least one, and possibly both, of these duplications arose after the chromosomes were placed in stock with the deficiency, confirming that copy-number variation arises frequently enough for such duplications to be a concern. This serves as a reminder that balancers kept in stock for long periods of time may carry unexpected structural variation that could affect experimental results.

By comparing WGS data of related balancer chromosomes, we can identify exchange events between a balancer and its normal sequence homolog. Balancers have previously been shown to experience SCO events within terminal, noninverted segments and DCO events within inverted segments (Miller *et al.* 2016a; b), and both types of exchange events were observed in this study as well (Figure 5). It is notable that, like exchange events that occurred between an internal region of the *3^rd^* chromosome balancer *TM3* and normal-sequence homologs (Miller *et al.* 2016a), the non-subtelomeric exchange events observed in this study occurred at least 2 Mb from the nearest inversion breakpoint. This strengthens the conclusion that inversion breakpoints suppress exchange over distances of 2–3 Mb.

Similar to observations of single exchange events in the distal unbalanced region of the *3^rd^* chromosome balancer *TM3*, the *2^nd^* chromosome balancer *CyO* allows exchange near the tip of *2R* (and may also allow single exchange events near the tip of *2L*). We therefore suggest that mutations in the intervals distal to 22D1 on *2L* and 58A4 on *2R* be balanced with *SM5*—not with *CyO*. (However, *SM6a* or *SM1* should work well to maintain mutations in the distal tip of *2R*.) Even though an SCO event was observed in the distal portion of *2R* on *SM5*, this extremely distal event occurred in the last 30–40 kb of the chromosome and affected only 5 genes. Thus, we still encourage the use of *SM5* for balancing distal genes. We also recommend maintaining more than one independent culture of stocks where there is risk of losing mutations from exchange with balancers.

Finally, WGS also provides an opportunity to investigate both the shared and unique SNPs affecting gene function that are carried by balancer chromosomes. Here, we were able to confirm or molecularly characterize 7 of the 9 previously reported marker alleles carried by these four *2^nd^* chromosome balancers (Table 4). In addition to known visible markers, all chromosomes carry a number of other mutations, most of which remain uncharacterized phenotypically. A study by Araye and Sawamura (2013) reported the presence of novel recessive lethal mutations carried by two balancers maintained in their lab. They suggested that WGS should reveal additional novel mutations affecting gene function. We identified 97 nonsense and splice site mutations (Table 5) shared among all four balancers studied (Table S7) as well as 26 nonsense and splice site mutations (Table 5) that are either unique among a family of balancers (Table S7) or unique to a specific balancer (Table S8). Knowing the genes that are mutated on balancers is important for researchers working with a specific gene who may not realize the balancer chromosome they are using is creating a heteroallelic loss-of-function genotype.

Whole-genome sequencing using short-read technology has now been completed for the Drosophila *X* chromosome balancers *FM7a* and *FM7c* (Miller *et al.* 2016b); the *2^nd^* chromosome balancers *SM1*, *SM5*, *CyO*, and *SM6a*; and the *3^rd^* chromosome balancers *TM3*, *TM6*, and *TM6B* (Miller *et al.* 2016a). These studies have revealed surprising findings about the structures of these chromosomes, the mutations carried by them, and the sequence diversity that exists among them. Sequencing a panel of presumably identical balancer chromosomes has also added to our understanding of the mechanisms by which inversion breakpoints suppress exchange, demonstrating that studies of these commonly used genetic tools can provide insights into challenging biological questions.

## Supplementary Material

Supplemental Material is available online at www.g3journal.org/lookup/suppl/doi:10.1534/g3.118.200021/-/DC1

## References

[bib1] Anderson J. A. Song Y. S. Langley C. H., 2008 Molecular population genetics of Drosophila subtelomeric DNA. Genetics 178(1): 477–487. 10.1534/genetics.107.083196 18202389 PMC2206096

[bib2] Araye Q. Sawamura K., 2013 Genetic decay of balancer chromosomes in *Drosophila melanogaster*. Fly (Austin) 7(3): 184–186. 10.4161/fly.24466 23648996 PMC4049851

[bib3] Bridges C. B., 1937 Correspondences between linkage maps and salivary chromosome structure, as illustrated in the tip of chromosome 2R of *Drosophila melanogaster*. Cytologia (Tokyo) Fujii Jubil(2): 745–755. 10.1508/cytologia.FujiiJubilaei.745

[bib4] Bridges C. B. Warren K. B., 1944 *The Mutants of Drosophila melanogaster*, Carnegie Institution of Washington, Washington, DC.

[bib5] Chen K. Wallis J. W. McLellan M. D. Larson D. E. Kalicki J. M., 2009 Breakdancer: an algorithm for high-resolution mapping of genomic structural variation. Nat. Methods 6(9): 677–681. 10.1038/nmeth.1363 19668202 PMC3661775

[bib6] Chintapalli V. R. Wang J. Dow J. A. T., 2007 Using FlyAtlas to identify better *Drosophila melanogaster* models of human disease. Nat. Genet. 39(6): 715–720. 10.1038/ng2049 17534367

[bib7] Cingolani P. Platts A. Wang L. Coon M. Nguyen T., 2012 A program for annotating and predicting the effects of single nucleotide polymorphisms, SnpEff: SNPs in the genome of *Drosophila melanogaster* strain *w^1118^*; *iso-2*; *iso-3*. Fly (Austin) 6(2): 80–92. 10.4161/fly.19695 22728672 PMC3679285

[bib8] Clark H. F. Brentrup D. Schneitz K. Bieber A. Goodman C., 1995 *Dachsous* encodes a member of the cadherin superfamily that controls imaginal disc morphogenesis in Drosophila. Genes Dev. 9(12): 1530–1542. 10.1101/gad.9.12.1530 7601355

[bib9] Cook R. K. Christensen S. J. Deal J. A. Coburn R. A. Deal M. E., 2012 The generation of chromosomal deletions to provide extensive coverage and subdivision of the *Drosophila melanogaster* genome. Genome Biol. 13(3): R21 10.1186/gb-2012-13-3-r21 22445104 PMC3439972

[bib10] Craymer L., 1980 New mutants report. DIS 55: 197–200.

[bib11] Craymer L., 1984 New mutants report. DIS 60: 234–236.

[bib12] Danecek P. Auton A. Abecasis G. Albers C. A. Banks E., 2011 The variant call format and VCFtools. Bioinformatics 27(15): 2156–2158. 10.1093/bioinformatics/btr330 21653522 PMC3137218

[bib13] Faust G. G. Hall I. M., 2014 SAMBLASTER: fast duplicate marking and structural variant read extraction. Bioinformatics 30(17): 2503–2505. 10.1093/bioinformatics/btu314 24812344 PMC4147885

[bib14] Graubard M. A., 1932 Inversion in *Drosophila melanogaster*. Genetics 17: 81–105. 17246645 10.1093/genetics/17.1.81 PMC1201651

[bib15] Hoskins R. A. Carlson J. W. Wan K. H. Park S. Mendez I., 2015 The Release 6 reference sequence of the *Drosophila melanogaster* genome. Genome Res. 25(3): 445–458. 10.1101/gr.185579.114 25589440 PMC4352887

[bib16] Hurd T. R. Liang F.-X. Lehmann R., 2015 *Curly* encodes dual oxidase, which acts with heme peroxidase Curly Su to shape the adult *Drosophila* wing. PLoS Genet. 11(11): e1005625 (erratum: PLoS Genet. 12(8): e1006285). 10.1371/journal.pgen.1005625 26587980 PMC4654585

[bib17] Kern A. D. Begun D. J., 2008 Recurrent deletion and gene presence/absence polymorphism: telomere dynamics dominate evolution at the tip of 3L in *Drosophila melanogaster* and *D. simulans*. Genetics 179(2): 1021–1027. 10.1534/genetics.107.078345 18505885 PMC2429855

[bib18] Kim N. Kim J. Park D. Rosen C. Dorsett D., 1996 Structure and expression of wild-type and suppressible alleles of the Drosophila *purple* gene. Genetics 142: 1157–1168. 8846895 10.1093/genetics/142.4.1157 PMC1207115

[bib19] Kurusu M. Cording A. Taniguchi M. Menon K. Suzuki E., 2008 A screen of cell-surface molecules identifies leucine-rich repeat proteins as key mediators of synaptic target selection. Neuron 59(6): 972–985. 10.1016/j.neuron.2008.07.037 18817735 PMC2630283

[bib20] Lewis E. B. Mislove R. F., 1953 New mutants report. DIS 27: 57–58.

[bib21] Li H. Durbin R., 2009 Fast and accurate short read alignment with Burrows-Wheeler transform. Bioinformatics 25(14): 1754–1760. 10.1093/bioinformatics/btp324 19451168 PMC2705234

[bib22] Li H. Handsaker B. Wysoker A. Fennell T. Ruan J., 2009 The Sequence Alignment/Map format and SAMtools. Bioinformatics 25(16): 2078–2079. 19505943 10.1093/bioinformatics/btp352 PMC2723002

[bib23] Lindsley D. L. Zimm G. G., 1992 *The Genome of Drosophila melanogaster*, Academic Press, San Diego, CA.

[bib24] Marygold S. J. Roote J. Reuter G. Lambertsson A. Ashburner M., 2007 The ribosomal protein genes and *Minute* loci of *Drosophila melanogaster*. Genome Biol. 8(10): R216 10.1186/gb-2007-8-10-r216 17927810 PMC2246290

[bib25] Miller D. E. Cook K. R. Arvanitakis A. V. Hawley R. S., 2016 a Third chromosome balancer inversions disrupt protein-coding genes and influence distal recombination events in *Drosophila melanogaster*. G3 (Bethesda) 6(7): 1959–1967. 10.1534/g3.116.029330 27172211 PMC4938649

[bib26] Miller D. E. Cook K. R. Yeganeh Kazemi N. Smith C. B. Cockrell A. J., 2016 b Rare recombination events generate sequence diversity among balancer chromosomes in *Drosophila melanogaster*. Proc. Natl. Acad. Sci. USA 113(10): E1352–E1361. 10.1073/pnas.1601232113 26903656 PMC4790991

[bib27] Mislove R. F. Lewis E. B., 1955 *SM5: Second Multiple 5*. DIS 29: 75.

[bib28] Morgan T. H. Bridges C. B. Schultz J. Schultz J., 1936 Constitution of the germinal material in relation to heredity. Year B. Carnegie Inst. Wash. 35: 289–297.

[bib29] Oster I. I., 1956 A new crossing-over supressor in chromosome 2 effective in the presence of heterologous inversions. DIS 30: 145.

[bib30] Perry T. McKenzie J. A. Batterham P., 2007 A *Dα6* knockout strain of *Drosophila melanogaster* confers a high level of resistance to spinosad. Insect Biochem. Mol. Biol. 37(2): 184–188. 10.1016/j.ibmb.2006.11.009 17244547

[bib31] Rozen S. Skaletsky H., 2000 Primer3 on the WWW for general users and for biologist programmers. Methods Mol. Biol. 132: 365–386. 10547847 10.1385/1-59259-192-2:365

[bib32] Sturtevant A. H., 1926 A crossover reducer in *Drosophila melanogaster* due to inversion of a section of the third chromosome. Biol. Zent. Bl. 46: 697–702.

[bib33] Sturtevant A. H., 1931 Known and probable inverted sections of the autosomes of *Drosophila melanogaster* In: *Contributions to the genetics of certain chromosome anomalies in Drosophila melanogaster*, Carnegie Institution of Washington, Washington, DC 421: 1–27.

[bib34] Ward L., 1923 The genetics of *Curly wing* in Drosophila. Another case of balanced lethal factors. Genetics 8: 276–300. 17246014 10.1093/genetics/8.3.276 PMC1200750

[bib35] Warren W. D. Palmer S. Howells A. J., 1996 Molecular characterization of the *cinnabar* region of *Drosophila melanogaster*: Identification of the *cinnabar* transcription unit. Genetica 98(3): 249–262. 10.1007/BF00057589 9204549

[bib36] Watson G. B. Chouinard S. W. Cook K. R. Geng C. Gifford J. M., 2010 A spinosyn-sensitive *Drosophila melanogaster* nicotinic acetylcholine receptor identified through chemically induced target site resistance, resistance gene identification, and heterologous expression. Insect Biochem. Mol. Biol. 40(5): 376–384. 10.1016/j.ibmb.2009.11.004 19944756

